# Aggravation of retinal hard exudates after intravitreal anti-vascular endothelial growth factor therapy for cystoid macular edema and the risk factors: a retrospective study

**DOI:** 10.1186/s12886-022-02315-z

**Published:** 2022-02-23

**Authors:** Rui Shi, Zhonglan Guo, Xiangxiang Yang, Xuanyi Che

**Affiliations:** Department of Ophthalmology, Shaanxi Provincial People’s Hospital, Xi’an, 710068 China

**Keywords:** DME, DR, BRVO, hard exudates, subretinal fluid, serum lipids

## Abstract

**Background/aims:**

To evaluate retinal hard exudates (HEs) progression in patients with cystoid macular edema (CME) secondary to diabetic retinopathy (DR) or branch retinal vascular occlusion (BRVO) after intravitreal injections of ranibizumab (IVR) treatment and identify the risk factors for the deterioration of HEs.

**Methods:**

This retrospective study enrolled 288 eyes with center-involving CME secondary to DR or BRVO from 288 patients (one eye per patient). All patients were treated with three loading doses of ranibizumab intravitreally at a monthly interval. The morphologic features of HEs were observed, and the HEs areas were quantified using a semi-automatic method at baseline, 1 month after the first dose of IVR and 1 month after the third dose of IVR therapy. HEs progression was defined as having a > =2-grade increase in the HEs severity scale. The best-corrected vision acuity (BCVA) and alterations in HEs areas were compared between DR and BRVO groups. And *logistic regression analyses* were used to identify the risk factors for HEs exacerbation.

**Results:**

Morphological changes of retinal HEs occurred in all eyes after IVR therapy, although HEs area was not significantly changed in some eyes. DR group has a higher percentage of eyes with progressed HEs area than the BRVO groups (34.9% vs. 21.8%, *P* = 0.019) 1 month after the first dose of IVR. Both DR and BRVO groups had a decreased percentage of enlarged HEs 1 month after the third injection, but the DR group is still higher than the BRVO group (17.1% vs. 8.4%, *P* = 0.027). At baseline, there was no correlation between VA and HEs areas. After the first and third doses of IVR, there still was no consistent correlation between HEs severity and change in VA over time. Furthermore, CME with subretinal fluid (SRF) is associated with a higher risk of HEs progression (*P* = 0.001). Long CME duration and high serum low-density lipoprotein cholesterol (LDL-C) level were identified as risk factors for HEs progression following IVR treatment in both *univariable* and *multivariable* regression analyses (Odds ratio (OR) = 1.88, *P* = 0.012 and OR = 1.14, *P* = 0.021, respectively).

**Conclusions:**

Alterations in the area of retinal HEs are widely observed after IVR treatment for CME. The eyes with CME secondary to DR have a higher percentage of progressed HEs than the BRVO eyes. DME with SRF, extended duration of CME, and high LDL-C level are potential risk factors of deteriorated HEs after IVR treatment.

## Introduction

Macular edema (ME) is a major vision-threatening complication in patients with diabetic retinopathy (DR) [1] or other retinal vascular disorders, such as retinal venous occlusion (RVO) [2]. In these ischemic retinal diseases, breakdown of the blood-retina barrier leads to increased vascular permeability of the macular capillaries and results in the various appearance of ME [3]. The morphologic features of ME in patients with DR or RVO are similar, including central retinal venous occlusion (CRVO) and branch retinal venous occlusion (BRVO) [4]. However, the visional outcome remains different between DR and RVO. One of the most common reasons is the occurrence of retinal hard exudates (HEs), which can be frequently observed along with ME [5]; profound central vision loss will occur when HEs congregate at the foveal center. Since it is difficult to improve visual acuity when outer retina structures of macular are destroyed by HEs, there is an urgent need to improve our understanding of the pathophysiologic mechanisms of HEs development and prevent it from reaching the foveal center.

Some studies have shown that intraocular injection of anti-vascular endothelial growth factor (VEGF) agents could provide substantial benefit to patients with ME secondary to DR and BRVO [6–9]. For example, monthly injections of ranibizumab have been reported to lead to an excellent outcome in a large proportion of patients with focal ME [10] and a greater reduction of HEs area (formed before the initial injection) compared with the sham therapy [11]. Another study also indicated that the presence and area of HEs did not increase as diabetic macular edema (DME) resolved (either in the ranibizumab or the sham group) [12]. But some researchers found that precipitation of HEs still can occur after the reduction of ME after an effective treatment [13] and hyperreflective foci, identified as the precursor of HEs, increased in size and areas at the early stage of treatment [14]. Therefore, it is of clinical significance to identify the causes of the deterioration of HEs after anti-VEGF therapy. Since the development of HEs is a complex process, many factors/mechanisms may be involved. The original diseases causing ME and HEs, such as DR and BRVO, may result in different outcomes of HEs after therapy. Special morphological features of ME may also be correlated to HEs deposit following anti-VEGF therapy. Some systemic risk factors, such as serum lipids [15] that play important roles in diabetic complications, may also be involved in the HEs development after anti-VEGF therapy. Notably, the underlying mechanism for the deterioration of HEs following anti-VEGF therapy is still largely unclear.

Macular cystoid edema (CME) with subretinal fluid (SRF) is the most prevalent type in the majority of patients with ischemic retinal vascular disease, espicially with DME. In CME, macular cysts result from a thickening of the outer nuclear layer when the volume of intraretinal fluid is increased due to impaired blood-retina barrier [16], and a localized retinal sensory detachment following the accumulation of SRF occurs when the external limiting membrane in the macular is affected. *Pemp* et al. [17] indicated that intraretinal aggregates of microexudates detectable as hyperreflective foci might compose and precede HEs before they become clinically visible, and the specific localization depends greatly on the presence of intraretinal fluid accumulation [18]. Thus, as the most common morphologic feature of ME, SRF is hypothesized to be associated with HEs deposit in patients with CME secondary to ischemic retinal diseases.

The aim of this study was to evaluate HEs progression in patients with CME secondary to DR or BRVO after intravitreal injections of ranibizumab (IVR) treatment. In addition, the systemic risk factors for the deterioration of HEs also were assessed.

## Materials and methods

### Participants and grouping

In this study, patients with center-involved CME secondary to DR or BRVO were enrolled from the Department of Ophthalmology in the Shaanxi Provincial Peoples’ Hospital. Data were collected from Jan.1, 2017, to Dec.30, 2020. Patients were diagnosed with type 2 diabetes by endocrinologists according to the following criteria: having a fasting plasma glucose level of more than 7.0 mmol/L or having symptoms of diabetes plus a casual plasma glucose concentration of more than 11.1 mmol/L. The diagnosis of DR and BRVO was made based on the results of the color fundus and fluorescein angiograms (FFA) by the same ophthalmologist.

All patients were administrated with a loading dose of three intravitreal injections of 0.5 mg ranibizumab (Lucentis®)at a monthly interval and followed up 1 month after the first and third dose of IVR respectively. The inclusion criteria are as follows. 1) patients aged 40 or older; 2) eyes with CME secondary to DR (any stage of NPDR with HEs) or superior temporal BRVO. The duration of the CME was determined by the self-reported duration of visual impairment. If both eyes of a diabetic patient had HEs, the right eye was enrolled; 3) CME with or without HEs in a 6 × 6 mm^2^ area of the macular; 4) CME with a center foveal thickness (CFT) more than 250 μm measured with spectral-domain optical coherence tomography (SD-OCT) [19]; 5) no intravitreal injections or laser photocoagulation in previous treatments within the past 6 months; 6) no prior history of vitreoretinal surgery, uveitis or other retinal diseases. Patients with significant media opacity or whose image quality strength was lower than 30 on SD-OCT were excluded. Based on the aims of this study, the enrolled eyes were divided into the DR group and BRVO group. All enrolled eyes were also grouped according to the HEs outcome after IVR therapy: eyes with HEs aggravation and eyes without HEs aggravation. The association between CME with pre-existing SRF and HEs aggravation was also evaluated.

### Baseline and follow-up eye examinations

Medical and ocular histories of all patients were collected from their medical records. Best-corrected visual acuity (with a standard Landolt C chart in a single room) was assessed at baseline and 1 month after the first and third dose of IVR respectively. Baseline and subsequent color fundus photographs were obtained from the storage of the Optos ultra-widefield (UWF) imaging systems (Optos Carfornia®, Optos PLC, Dunfermline, United Kingdom). FFA was graded at the clinic of the Shaanxi Provincial People’s Hospital. The OCT imaging system (3D OCT-1 (ver.8.30); Topcon Corporation, Japan) was used to measure CFT, which was defined as the average retinal thickness in the central subfield of a standard ETDRS grid. The standardized macular cube protocol scan (512 × 128) consists of 128 horizontal B-scan lines, each composed of 512 A-scans over a 6-mm square that was centered on the fovea. CFT was automatically calculated using the built-in software. SRF was defined as the space between the RPE layer and the neurosensory retina. Color fundus photographs were used to mask all the HEs regions in the macular and the areas of HEs were quantitatively measured with semi-automated imaging software. The area of macular HEs and the numbers of eyes with SRF were compared between groups at baseline. All the examinations except FFA were also conducted at one- and three-month follow-up after ranibizumab injections.

### Quantitative measurement of HEs area

**Fig. 1:**
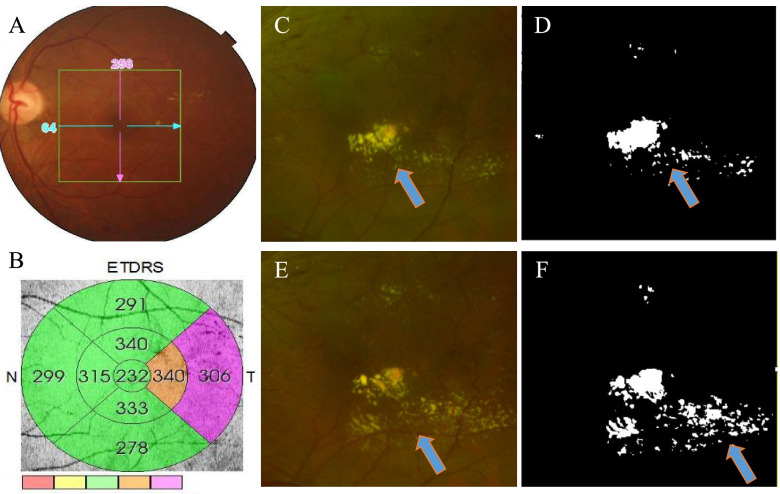
Quantitative measurement of the area covered by HEs. **A** A colorful fundus photograph cropped for a 6 × 6 mm^2^ square area; **B** An ETDRS grid for determining HEs locations; **C** Representative macular HEs in a colorful fundus photograph at baseline; **D** Area covered by HEs is calculated by quantifying the over-threshold (white color) area using the automatic thresholding function of Image J. **E** Macular HEs in the same patients at the end of the first month following IVR therapy. **F** The HEs area is increased compared to the baseline area by quantifying the white color region. Blue arrows indicate the HEs deposits

Quantitative measurement of HEs area was performed using Image J (version 1.52p; http://rsb.info.nih.gov/ij//; National Institutes of Health, Bethesda, MD, USA) [20]. Because of the variability in fundus photography magnification due to corneal curvature, axial length, refractive error, and depth of focus, we examined the HEs area in a 6x6mm square that was centered on the fovea, which was matched with a standard ETDRS grid (Fig. 1A and B). Firstly, the cropped images were split into three color channels, and the green channel was used for the analysis because it highlights HEs pathology very well. Then, we set the scale for the macular picture as millimeter (mm) instead of pixels according to the 6x6mm exact macular area. Thirdly, we used the “Maxentropy” function in ImageJ to identify HEs using an intensity threshold that was set to maximize the capture of all possible HEs in the macular. After that, two masked investigators were employed to evaluate the images and decide independently whether the automated function had missed or incorrectly identified HEs and manually corrected the images. Finally, the HEs area for each cropped image was measured automatically in millimeters using the “measure” function of ImageJ. We calculated a relative area of a HEs by dividing the HEs area by the total cropped image area (Fig. 1 C-F). The area of HEs was graded as absent, minimal (> 0–0.1 mm^2^), mild (> 0.1–0.5 mm^2^), moderate (> 0.5–2.5 mm^2^), and severe (> 2.5 mm^2^). Aggravated HEs were defined as having a > = 2-grade increase in the HEs severity scale (for example, increased from minimal to moderate) [12].

### Body composition parameters and laboratory tests

The demographic characteristics, serum lipid profile, including total cholesterol (TC), triglycerides (TG), LDL-C, and high-density lipoprotein cholesterol (HDL-C) levels, were collected from the medical records of the patients.

### Statistical analysis

Statistical analyses were conducted using GraphPad Prism 8 (GraphPad Software Inc., USA). Data were presented as *mean ± standard deviation (SD)* of each group. The baseline characteristics and OCT parameters were compared using an *independent-sample t-test* for numerical values and *a chi-square test* on 2 × 2 contingency tables for discrete variables. A *Chi-square test* was also performed to determine the association between SRF involving the central macula and the presence of 2-grade HEs aggravation at the end of the first month after IVR. *Univariate* and *multivariable logistic regression* analyses were performed to identify the potential risk factors for HEs development. In the regression assays, the presence of HEs aggravation was the dependent variable, and the independent variables included age, gender, CME duration, CFT, HEs area and serum lipids levels. For all tests, a *P < 0.05* was considered statistically significant.

## Results

### Baseline characteristics of the patients

According to the inclusion and exclusion criteria, 288 eyes from 288 patients with center-involving CME secondary to DR (146 eyes) or BRVO (142 eyes) were enrolled in this retrospective study. No significant difference was discovered in age, gender, duration of CME, BCVA, CFT, HEs area, number of eyes with SRF, TC, TG, LDL-C, and HDL-C between the DR and BRVO groups. The baseline characteristics of the enrolled patients and eyes are thus comparable between the two groups and are summarized in Table 1.

**Table 1:** Clinical characteristics of patients with DR and BRVO

	DR	BRVO	*p*-value
Participants/Eyes (n)	146	142	–
Gender (male %)	52.0	48.0	–
Age (yrs)	55.34 ± 14.77	56.94 ± 19.28	0.497
BCVA (logMAR) (mean ± SD)	0.53 ± 0.21	0.55 ± 0.13	0.333
CME duration (m)	7.61 ± 8.13	5.77 ± 3.14	0.172
CFT (μm)	532.24 ± 76.33	545.55 ± 80.97	0.152
CME without SRF	69 (47.2%)	78 (54.9%)	0.240
CME with SRF	77 (52.7%)	64 (45.0%)	
HEs area (mean ± SD)	0.61 ± 0.35	0.55 ± 0.58	0.287
HEs severity			
Absent	38 (26.0%)	41 (28.9%)	0.704
Minimal (> 0–0.1 mm^2^)	44 (30.1%)	47 (33.1%)	0.719
Mild (> 0.1–0.5 mm^2^)	33 (22.6%)	29 (20.4%)	0.780
Moderate (> 0.5–2.5 mm^2^)	23 (15.8%)	18 (12.7%)	0.616
Severe (> 2.5 mm^2^)	8 (5.5%)	7 (4.9%)	0.842
Laboratory data			
Total cholesterol (mmol/L)	4.41 ± 0.96	4.69 ± 0.88	0.231
Triglyceride (mmol/L)	1.33 ± 0.48	1.36 ± 0.32	0.575
LDL-C (mmol/L)	2.26 ± 0.21	2.19 ± 0.53	0.226
HDL-C (mmol/L)	1.09 ± 0.17	1.15 ± 0.43	0.201

*BCVA* Best correction vision acuity, *BRVO* Branch retinal vein occlusion, *CFT* Center foveal thickness, *CME* Cystoid macular edema, *DR* Diabetic retinopathy, *HDL* High-density lipoprotein. *LDL* Low-density lipoprotein. Data represent the mean ± SD of each group

### Outcomes and morphological changes of retinal HEs following IVR therapy

**Fig. 2:**
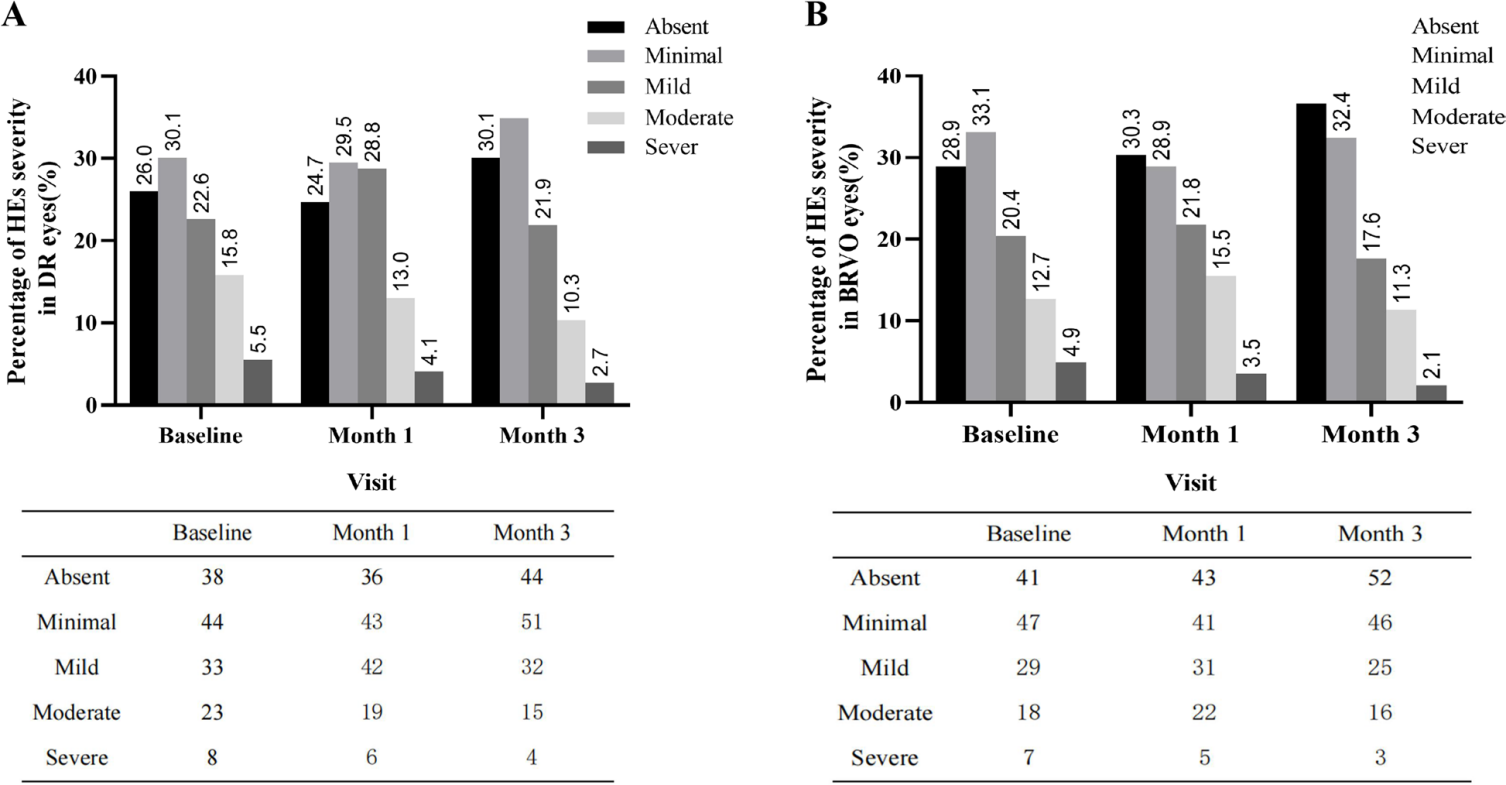
Hard exudates severity at baseline, month 1 and month 3. The figure shows the percentage of eyes in each category in both (**A**) DR and (**B**) BRVO groups. Hard exudate severity categories were defined according to Early Treatment Diabetic Retinopathy Study criteria as absent (no hard exudate present), minimal (> 0–0.1 mm^2^), mild (> 0.1–0.5 mm^2^), moderate (> 0.5–2.5 mm^2^), and severe (> 2.5 mm^2^)

**Fig. 3:**
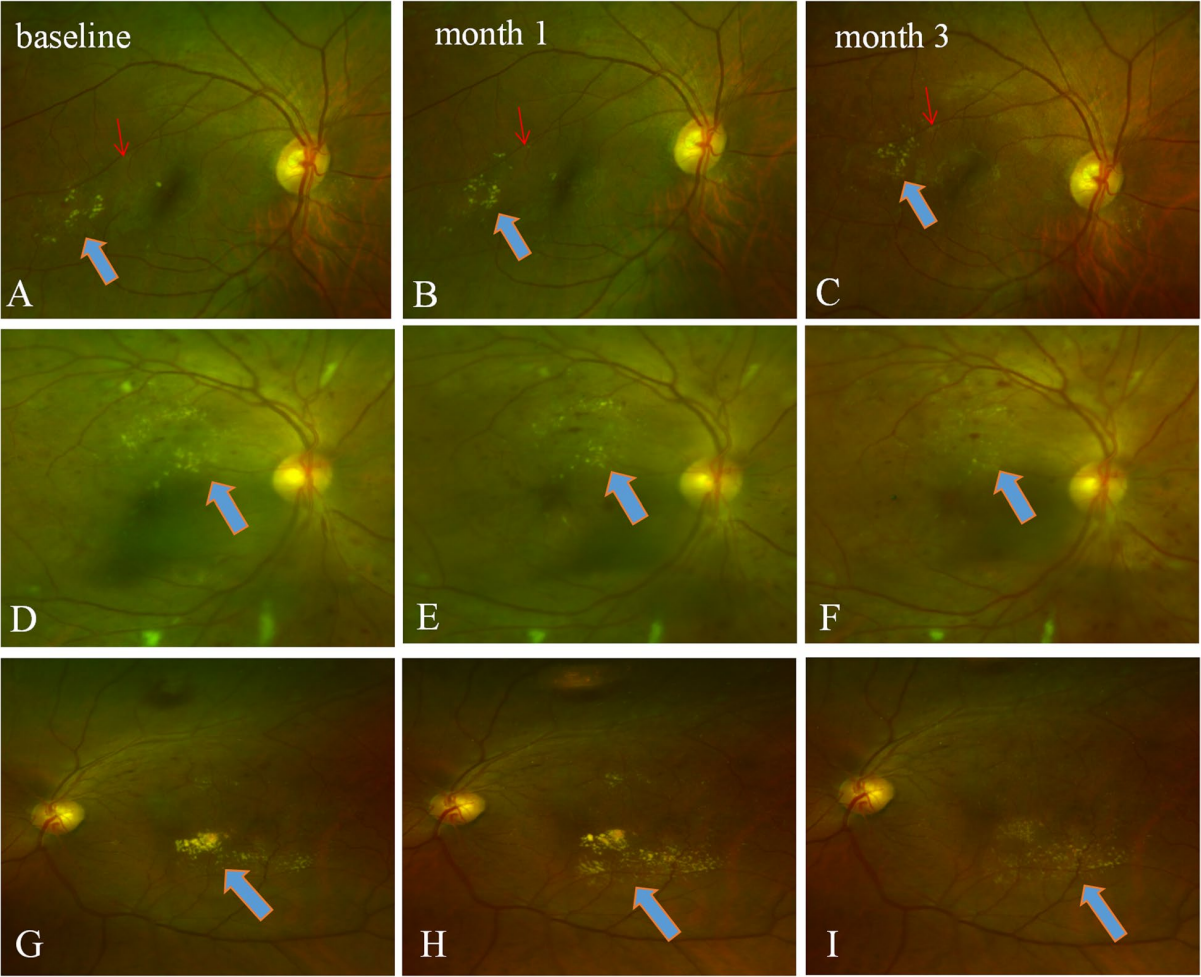
Fundus photographs acquired before and after the IVR therapy. (A-C) HEs area remains the same after IVR; however, the locations of HEs are changed (refer to the blood vessel marked with a red arrow). **A** baseline; **B** one month after IVR therapy; **C** three months after IVR therapy. **D**-**F** HEs area is reduced after IVR therapy. **D** baseline; **E** one month after IVR therapy; **F** three months after IVR therapy. **G**-**I** HEs area is enlarged at one month but partly decreased at three months after IVR therapy. **G** baseline; **H** one month after IVR therapy; showing HEs area is increased when compared with baseline area; **I** three months after IVR therapy, showing the HEs area is smaller than that of one month. Blue arrows indicate the HEs deposits

The number, density and location of macular HEs were observed in the fundus photographs of all 288 eyes, and the area of HEs before the treatment and at one- and three-month follow-up examinations was calculated (Fig. 2). HEs were clinically visible in part of enrolled eyes before the treatment (Fig. 3A, D, and E). At month one after IVR, although HEs areas remained unchanged in 143 eyes (49.6%), the location, number and density of HEs were changed in these eyes (Fig. 3A-C); 69 eyes (23.9%) showed a 2-grade reduction in HEs area (Fig. 3D-F); enlarged areas of HEs, defined as having > = 2-grade aggravation, were observed in 76 eyes (26.3%) (Fig. 3G-I, Table 2). However, in the eyes with aggravated HEs, the HEs deposit was significantly reduced at the end of the third month after IVR. The number of eyes with > = 2-grade reduction in HEs was increased to 155 eyes (53.8% vs. 23.9% at month one, *P* < 0.001), while the number of eyes with > = 2-grade aggravation of HEs was decreased to 43 (14.9% vs. 26.3% at month one) (Table 2). These findings suggest that HEs are dynamically changing and may progress in some eyes after IVR therapy, which is a short-term process and may resolve as the duration after treatment is elongated.

**Table 2:** Outcomes of macular HEs following IVR therapy

	One month	Three month	***P-value***
*n*	288	288	
2-grade reduction	69 (23.9%)	155 (53.8%)	0.000^***^
No change in the area	143 (49.6%)	90 (31.2%)	0.001^**^
2-grade enlargement	76 (26.3%)	43 (14.9%)	0.000^***^

*IVR* Intravitreal injections of ranibizumab. **, *p* < 0.01; ***, *p* < 0.001, compared one month and three month data

### The difference in retinal HEs area between the DR and BRVO groups

The baseline retinal HEs areas of eyes with center-involved CME were similar in the DR and BRVO groups (*P* > 0.05, Table 1). At the end of the first month after IVR, the DR group showed a significantly higher rate of > = 2-grade HEs aggravation than the BRVO group (34.9% vs. 21.8%, *P* = 0.018). Both groups showed a much lower HEs aggravation rate at the end of the third IVR, and the difference became smaller (17.8% vs. 8.4%, *P* = 0.027). These results suggest that DR patients are more likely to have deteriorated HEs than BRVO patients after IVR treatment, but the progression of HEs is temporary and likely resolved in several months after the treatment.

### Relationship between pre-existing SRF and HEs progression after IVR therapy

**Fig. 4:**
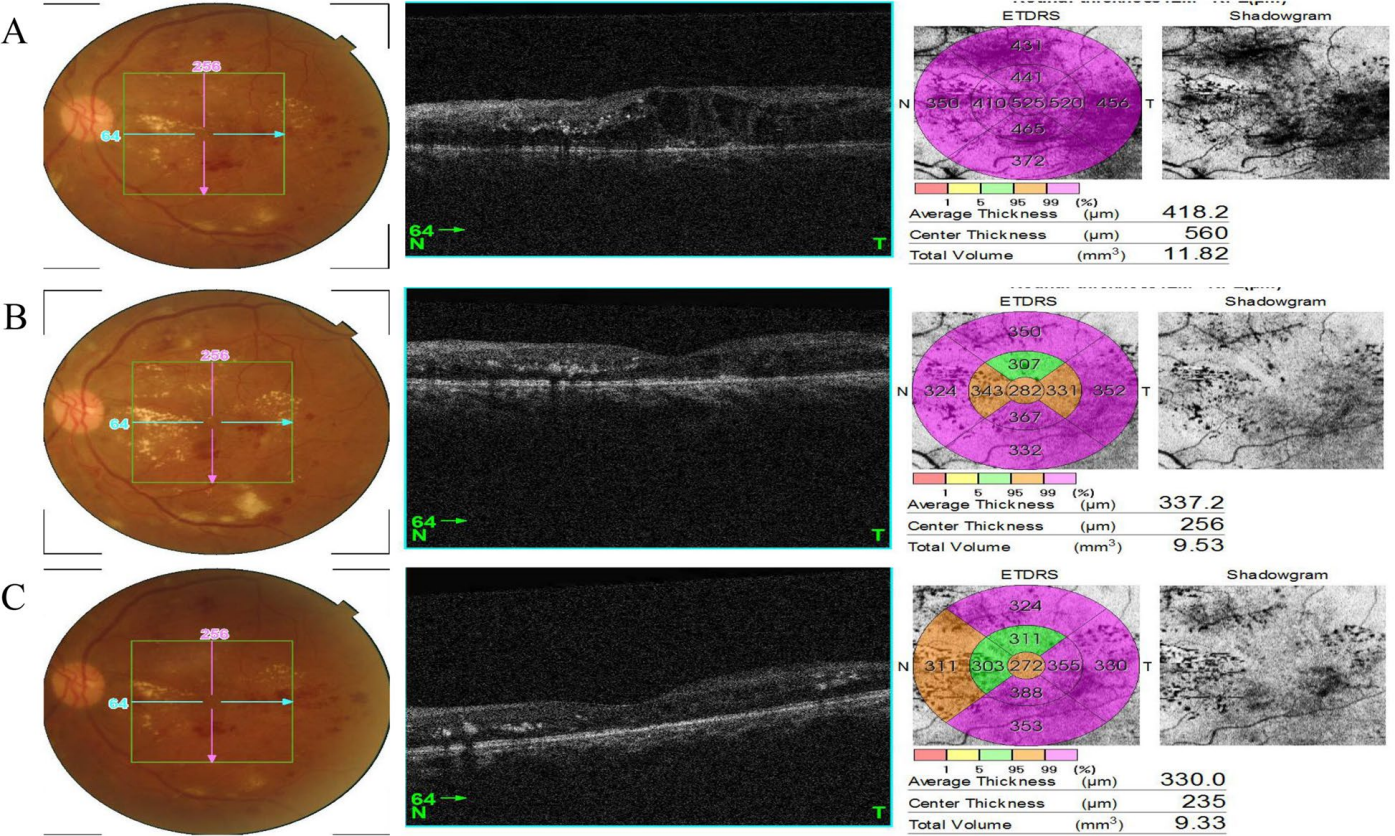
Representative OCT images show the dynamic changes of HEs in eyes with SRF-free CME following IVR treatment. **A** An eye with CME and HEs at baseline; **B** Shrunk CME with increased HEs area at the end of one month; **C** HEs are partly resolved when the follow-up is extended to three months. For A-C, the left panel is the fundus photograph showing HEs in the green square, the middle panel is a vertical cross-section of retinal layers showing macular edema, the right panel shows the ETDRS grid showing macular thickness

**Fig. 5:**
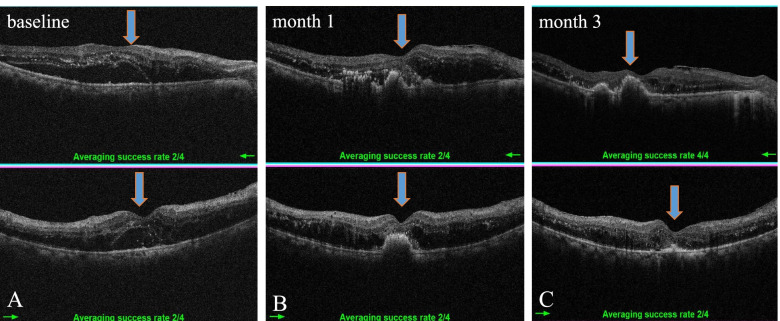
Representative SD-OCT photographs show HEs dynamic changes in eyes with SRF-positive CME secondary to BRVO following IVR treatment. **A** An eye with CME, SRF, and HEs at baseline; **B** Shrinked CME with increased HEs area at the end of one month; **C** HEs area is partly decreased when follow-up was extended to three months after IVR. For A-C, the left panel is the fundus photograph showing HEs in the green square, the middle panel is a vertical cross-section of retinal layers showing macular edema, the right panel is the ETDRS grid showing macular thickness

**Fig. 6:**
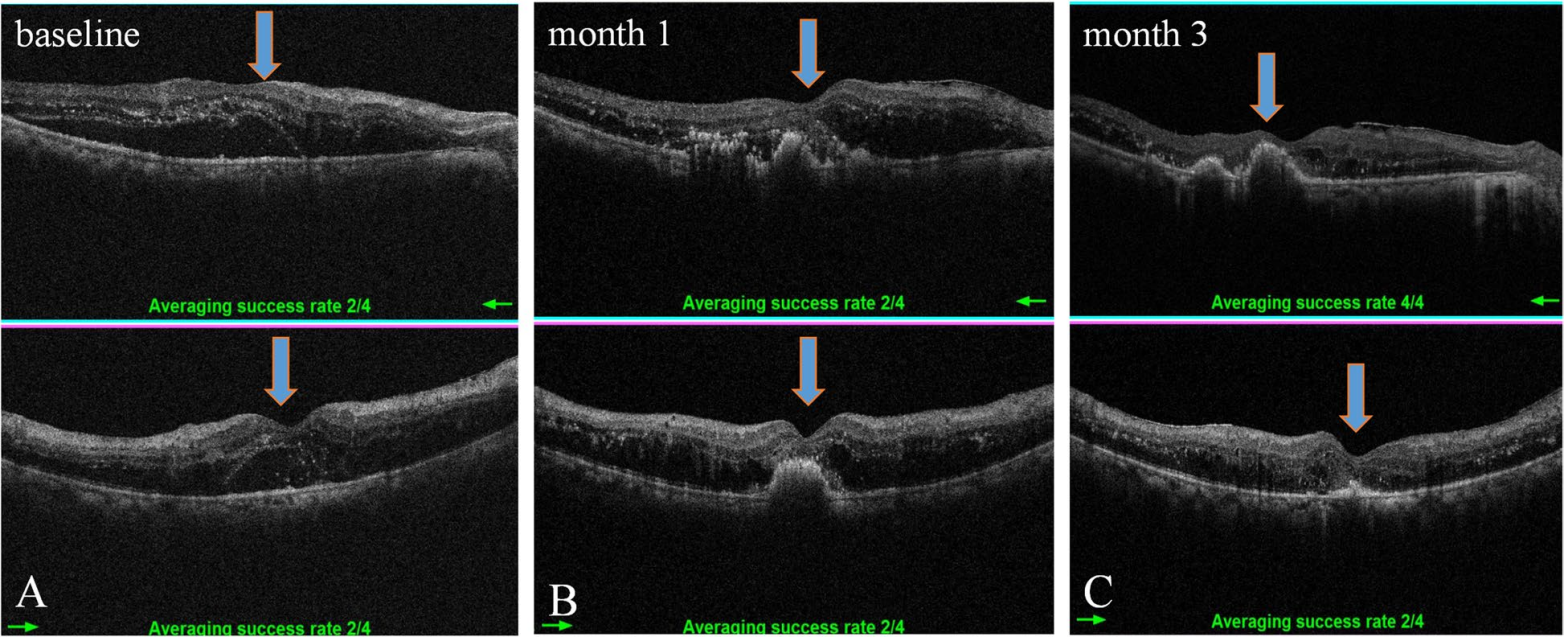
Representative SD-OCT images acquired at **A** baseline, **B** one month, and **C** three months after IVR therapy show HEs appeared at corresponding locations of SRF during the follow-up period after IVR injections. Blue arrows indicate the HEs deposits. The upper panel indicates horizontal scanning of macular, low panel shows vertical scanning of the same macular

According to the SD-OCT images, a rapid reduction in macular edema occurred in all enrolled eyes in the first month after initial treatment. Since CME with SRF is the most prevalent type of macular edema in patients with DME and RVO, SRF is supposed to be associated with HEs deposit in ischemic retinal diseases, we then evaluated the association between the pre-existing SRF and post-IVR HEs progression based on the SD-OCT images at the end of the first-month following IVR. We found that increase in areas of HEs might occur in eyes with (Fig. 4) or without SRF (Fig. 5). However, the prevalence of aggravated HEs in SRF-free eyes is significantly lower than that of eyes with SRF (18.3% vs. 34.7%). In addition, macular HEs aggravation was more likely to occur in patients with DME accompanied by SRF rather than that of BRVO (44.1% vs. 23.4%) (Table 3). Furthermore, HEs are located at corresponding locations of SRF on fundus photographs (Fig. 6A-C).

**Table 3:** Association of SRF and two-grade macular HEs aggravation at one month in both groups

	Disease	***n***	HEs aggravation eyes	HEs non-aggravation eyes	***P***-***value***
CME without SRF	DR	69	17 (24.6%)	52 (75.3%)	0.087
	BRVO	78	10 (12.8%)	68 (87.1%)	
CME with SRF	DR	77	34 (44.1%)	43 (55.8%)	0.010*
	BRVO	64	15 (23.4%)	49 (76.5%)	
Total	–	288	76 (26.3%)	212 (73.7%)	
CME without SRF	–	147	27 (18.3%)	120 (81.6%)	0.001*
CME with SRF	–	141	49 (34.7%)	92 (65.2%)	

*HE* Hard exudate, *CME* Cystoid macular edema, *SRF* Subretinal fluid. *, *p* < 0.05 by *chi-square test*

### Relationship of HEs severity scale and BCVA

**Fig. 7:**
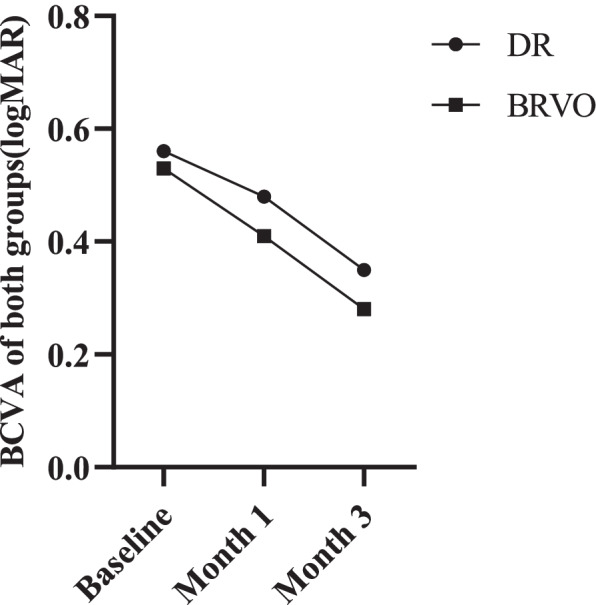
The mean BCVA changes in patients with DR or BRVO at different time points

No significant difference in BCVA was found between different groups at baseline (*P* = 0.333). Meanwhile, no significant correlation between baseline BCVA and the total area of HEs was discovered, and the Pearson correlation coefficients in DR and BRVO groups were − 0.665 (*P* = 0.061) and − 0.057 (*P* = 0.882), respectively. As the follow-up period was extended to 1 month after the third IVR injection, there was still no positive relation between HEs areas and BCVA (Fig. 7)**.**

### Identification of systemic risk factors for HEs progression

To identify the risk factors for HEs aggravation after IVR therapy, we carried out *univariate* and *multivariable logistic regression* analyses, adjusting for age and gender. The *univariate* regression identified high TC and LDL-C levels and a long CME duration as risk factors of HEs development following IVR treatment (all *P < 0.05*) (Table 4). The *multivariable logistic regression* assay further validated that a high LDL-C level and a long CME duration were risk factors of HEs progression (both *P < 0.05*) (Table 4). These results suggest that lowering blood LDL-C level and early management of CME may prevent the progression of HEs after IVR therapy.

**Table 4:** Risk factors of HEs aggravation following IVR by Logistic regression analysis

Risk factors	Univariate		Multivariate	
	OR (95% CI)	***P-value***	OR (95% CI)	***P-value***
TG (mmol/L)	1.04 (0.72–1.44)	0.753	1.03 (0.92–1.14)	0.621
TC (mmol/L)	**1.08 (1.01–1.28)**	0.045*	1.06 (0.65–1.77)	0.320
HDL-C (mmol/L)	0.87 (0.44–1.79)	0.268	0.62 (0.10–1.92)	0.601
LDL-C (mmol/L)	**1.37 (1.06–2.01)**	0.001**	**1.14 (1.03–1.97)**	0.021*
Duration of CME(m)	**1.98 (1.45–2.23)**	0.046*	**1.88 (1.01–2.75)**	0.012*
CFT (μm)	2.17 (1.59–3.77)	0.344	2.01 (1.42–3.29)	0.384
HEs severity	1.02 (0.85–1.88)	0.319	0.99 (0.51–1.83)	0.337

*HEs* Hard exudates, *IVR* Intravitreal injections of ranibizumab, *TG* Triglycerides, *TC* Total cholesterol, *HDL* High-density lipoprotein, *LDL* Low-density lipoprotein, *SRF* Subretinal fluid, *CME* Cystoid macular edema, OR Odds ratio, *CI *Confidence interval. *, *p* < 0.05; **, *p* < 0.01. A *p* < 0.05 was defined as statistically significant

## Discussion

CME combined with HEs can cause severe vision loss in patients with DR or BRVO. IVR could provide substantial benefits to patients with CME. However, the effect of IVR on macular HEs remains controversial. In the current study, we observed the morphological changes of HEs in patients with different diseases in the short term after IVR to identify relevant factors for explaining these clinical observations. The results demonstrated that the reduction in CME was accompanied by three different changes (reduced, unchanged and increased) in the areas of HEs that existed before the treatment in both DR or BRVO groups. Compared with the BRVO group, eyes with DME had a continuous increase in the area of HEs in the first month of therapy, which might gradually reduce in the following 2 months. We also found that the pre-existing SRF in the macular before intravitreal ranibizumab was associated with HEs aggravation compared with eyes without SRF at baseline. In addition, higher LDL cholesterol and longer duration of CME were identified as risk factors for HEs aggravation after anti-VEGF therapy.

According to the results from a randomized clinical trial in patients with DME, monthly intravitreal ranibizumab treatment has been proved to be beneficial for the reduction of HEs area in most eyes at the end of the 24-month follow-up period. However, some eyes still had a worsening of HEs at any time during the study [12], but the mechanism is unclear. Another study described the dynamic changes in hyperreflective structures [21], which is proved to be the precursors of HEs, during anti-VEGF treatment for DME and reported that microexudates detected as hyperreflective foci undergo a reorganization within a few days after the first treatment, including downward shift and aggravation of hyperreflective spots [17]. However, no automatic software is available for HEs measurement to evaluate the outcomes during IVR treatment. In the present study, we observed the morphologic changes of HEs in areas in the macular, using a semi-automatic quantitative measurement software; the results indicate that a significant resolution of HEs in 23.9% of eyes in all enrolled eyes, and it raised at the third months. Some eyes remain unchanged in the areas of macular HEs, and others (26.3%) in both treatment groups had a worsening of HEs at the first month following IVR; however, it declined to a low level at the third month. According to the previous studies, the treatment for DME can leave residual HEs as an extracellular precipitate within the retina as the macular edema resolves [22]. Fluid resorption by the retinal pigment epithelium results in a constant flow toward outer retinal segments, and macromolecules may be persisting and accumulating in the outer retina as the clearance of large osmotically active molecules. We speculated that DME reduction is accompanied by rearrangement of microexudates in the retina after IVR. The HEs outcomes are related to the balance of microexudates absorption and deposition while CME is declining. The aggravation of HEs in the short term after IVR may lead to limited vision improvement after CME treatment with IVR, so there is an urgent need to improve our understanding of the pathophysiologic mechanisms of HEs development after IVR.

Another unique feature of this study is that it compared the HEs progression rate in eyes with CME secondary to DR and BRVO during the follow-up period. The results showed that no significant difference was found in the areas of HEs between the two groups at baseline. However, a higher prevalence of enlarged HEs in eyes with DR was observed compared to those with BRVO at the end of the first-month follow-up, although they had similar morphological features on the OCT test. There was a decrease in the area of HEs in both groups in the next 2 months, and the difference between DR and BRVO groups became smaller. These results suggest that DR patients more likely to have aggravated HEs than BRVO patients after IVR treatment, but the progression of HEs is temporary and resolved several months after treatment. The results of the current study are in agreement with a previous report by *Pemp* et al. [17].

The breakdown of the inner and outer blood-retinal barrier (BRB) is the hallmark of ischemic retinopathy, which results in hyperpermeability of retinal vessels and causes CME. According to the results of the present study, we hypothesize that prolonged hyperglycemia may result in retinal inflammation in patients with DR, which is responsible for the deregulation of the endothelial cells of retinal vessels (inner BRB) as well as the retinal pigment epithelium (RPE) cell junction proteins (outer BRB) and impacts the blood supply of choroid. Then the poor RPE pumping capacity and impairment of choroidal blood flow limit the migration of water and lipid from the outer retina to the choroid in diabetic patients. Hence, HEs formation, enlargement, and deposition are more likely seen in the macular foveal accompanied by a rapid DME decline. However, CME secondary to acute BRVO seems to be related to inner retinal vascular (supplied by the central retina system) leakage, which is caused by ischemia-induced hypoxia rather than retinal inflammation and leads to an enhanced lipid and protein exudation. The relative normal blood supply of RPE cells by ciliary artery permits a more rapid effect on the regression of HEs in patients with CME secondary to BRVO when compared with DR. Hence, it seems that patients with DME are more likely to suffer from massive HEs deposition in the foveal during anti-VEGF therapy.

Different hypotheses regarding the pathophysiology of HEs deposition have been proposed for patients with DME [23, 24]. The SRF is visible on OCT as a hyporeflective area beneath the neuroretina. Leakage from the retinal or choroidal circulation into the subretinal space that exceeds reabsorption capacity is thought to be the main mechanism. Several studies reported that DME accompanied by SRF might be associated with the development of hyperrefective foci, which represents microexudate deposits of extravasated plasma lipids and/or proteins that might precede and compose HEs. However, controversies remain for the origin of hyperrefective foci to be precursors of HEs [25, 26]. So the relationship between SRF and deposition of HEs in the macular remains unclear. Among the 288 eyes in the current study, a significant aggravation of HEs was seen in 76 eyes in both groups at the end of the first month after IVR. The data indicated that eyes with SRF in the macular more likely to have HEs exacerbation in the first month of anti-VEGF therapy when compared to the ones without SRF. In addition, HEs in eyes with DME accompanied by SRF are more likely enlarged after IVR therapy when compared to BRVO. The present study indicates that the HEs area is significantly enlarged within the early stage after treatment and then gradually decreases in the following 3 months after IVR treatment in patients with SRF. We, therefore, hypothesize that anti-VEGF therapy contributes to fluid resorption by regulating the activity of the retinal pigment epithelium to allow a constant flow toward outer retinal segments. However, plasma lipids and proteins extravasated from the retinal capillaries cannot rapidly penetrate the external limiting membrane, which is acting as a transport barrier of large osmotically active molecules. The slow drainage of retinal capillaries exudates and poor RPE pumping capacity together cause the accumulation of fluids in the outer retina. Therefore, HEs aggravation was observed in the first month. However, over a long period of time after anti-VEGF treatment, the alleviated retinal inflammation and restored RPE cell activity leads to HEs absorption.

In this study, we also investigated the systemic risk factors for HEs progression. We found that a higher LDL level was associated with HEs aggravation after therapy; this result is consistent with that of a clinical trial [27]. Our previous studies reported that lipid-lowering agents are effective on HEs reduction [28, 29]; therefore, strict control of serum lipid might be an effective treatment for HEs therapy. In addition, we also found that a longer duration of CME was associated with an increased risk of HEs deposition in the macular during the follow-up period by a multivariate regression model. We speculated that a long duration of macular edema might increase the concentrations of inflammatory cytokines and VEGF in the vitreous humor, which was significantly associated with the presence of SRF [30], while more new microexudates deposit in the intraretinal of macular with a rapid decrease of retinal thickness and formed clinical HEs.

There are some limitations to our study that should be noticed. Firstly, oral lipid-lowering agents treatment in patients with diabetes and dyslipidemia is effective for HEs reduction and subfoveal lipid migration in CME [31]; however, we did not take into account oral administration of lipid-lowering agents when assessing the effect of anti-VEGF agents on HEs development. Secondly, this is a retrospective study; only superior temporal BRVO was enrolled in the present study; however, the underlying mechanism of HEs development might become different when different retinal branch vessels are blocked. Thirdly, CME duration was determined by the self-reported duration of visual impairment, which was thought subjective and often unreliable. That may impact the results of this study. In addition, because of the relatively short follow-up period and the various treatment regimens for CME, further prospective studies with a long observation period are needed to elucidate the difference of HEs deposition with ME secondary to various retina diseases.

## Conclusions

In sum, HEs aggravation in CME eyes with SRF was more pronounced in diabetic patients, especially in the first months following IVR therapy. In addition, a high LDL-C level and a long duration of CME are risk factors of HEs aggravation. Since this study has a short follow-up duration, longer follow-up periods are needed to evaluate the effect of IVR treatment on controlling HEs in diabetic patients and to identify the risk factors for deteriorated HEs following the IVR therapy.

## Data Availability

The original datasets and analyses are available from the corresponding author upon reasonable request.

## Abbreviations

DM: Diabetic mellitus
DR: Diabetic retinopathy
BCVA: Best-corrected vision acuity
BRVO: Branch retinal vein occlusion
CME: Cystoid macular edema
VEGF: Vascular endothelial growth factor
SRF: Subretinal fluid
HEs: Hard Exudates
HbA1c: Glycated hemoglobin
HDL: High-density lipoprotein
LDL: Low-density lipoprotein
AL: Axial length
IOP: Intraocular pressure
